# Microcomputed tomography used to link head morphology and observed feeding behavior in cichlids of Lake Malaŵi

**DOI:** 10.1002/ece3.7359

**Published:** 2021-03-30

**Authors:** Adrianus F. Konings, Joshua M. Wisor, Jay R. Stauffer

**Affiliations:** ^1^ Cichlid Press El Paso TX USA; ^2^ Ecosystem Science and Management Penn State University University Park PA USA; ^3^ South African Institute for Aquatic Biodiversity Makhanda South Africa

**Keywords:** cichlids, feeding behavior, head morphology, Lake Malaŵi, microcomputed tomography

## Abstract

Cichlids inhabiting the Great Lakes of Africa have radiated extremely rapidly, with Lake Malaŵi harboring some 850 species. This rapid radiation may be linked to the diversity in behaviors, sexual selection, and phenotypic plasticity. To determine the relationships between morphology and behaviors, microcomputed tomography (microCT) was used to observe internal morphological structures. Observed morphological adaptations were linked with observed behavior of cichlids in Lake Malaŵi with respect to the various available food resources. Many of these adaptations have parallels, sometimes into the finest details, in other drainage systems and can thus be considered as variations of how cichlids in general respond to environmental opportunities and challenges. Variations in the structure and teeth of the pharyngeal jaws and the oral jaws allowed for fine tuning of specializations, so that various species can utilize the same source without direct competition. We suggested that high‐resolution X‐ray computed tomography will permit scientists to infer life history and behavior characters of rare or extinct taxa from a detailed examination of morphology and linkages between morphology and behavior found in extant species.

## Graphical abstract

We linked observed morphological traits with behavioral observations of fishes from Lake Malawi. We used microcomputed tomography to obtain morphological traits and SCUBA to record behavior.

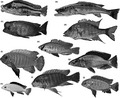

## 1 INTRODUCTION

Cichlids are best known for their explosive speciation, unique feeding specializations, and diverse mating systems (Stauffer et al., 2007) all of which allow them to adapt rapidly to new environments. Adaptive radiations contribute to the biodiversity of freshwater fishes as shown in the *Telmatherina*, a genus of freshwater fish from Indonesia, in which three lineages that exploited different resources could be distinguished by skull shape and pharyngeal bone configurations (Roy et al., 2007). Wainwright et al. (2005) introduced the concept of many‐to‐one mapping and suggested that there is a morphological basis for an organism's performance. The intercorrelation of performance traits may provide knowledge of relationships between structure and function (Vanhooydonck et al., 2006). Certainly, lineages that are comprised of diverse parts may have the potential to be morphologically diverse and result in the evolution of structure and function (Vermeij, 1973). Stauffer and Snik‐Gray (2004) suggested that phenotypic plasticity led to the trophic diversity and radiation of fishes in Lake Malaŵi.

**FIGURE 1:**
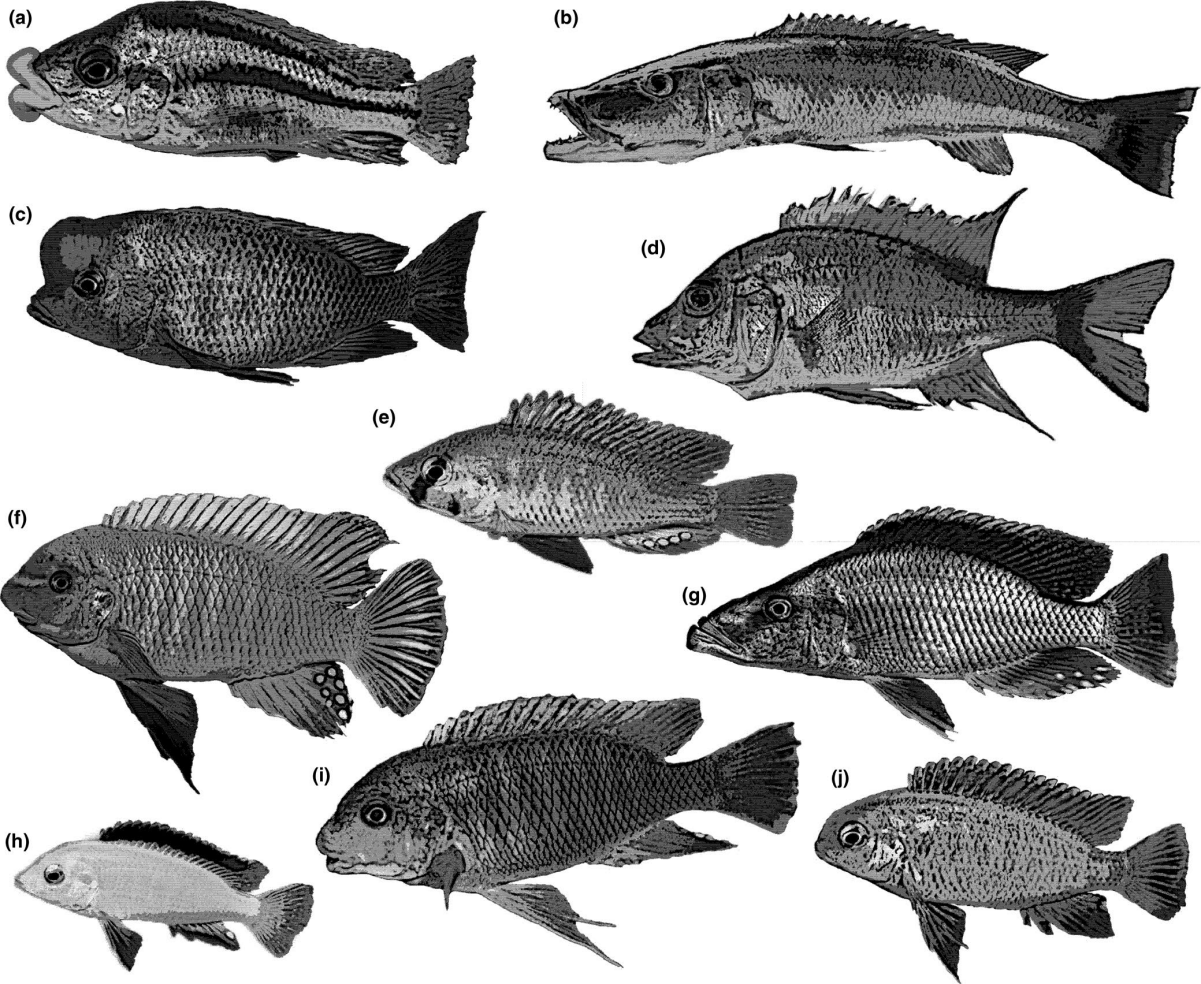
Variation in body morphology of cichlid fishes found in Lake Malaŵi. a. *Chilotilapia euchilus*, b. *Rhamphochromis esox*, c. *Cyrtocara moorii*, d. *Alticorpus macrocleithrum*, e. *Astatotilapia calliptera*, f. *Labeotropheus fuelleborni*, g. *Dimidiochromis compressiceps*, h. *Labidochromis caeruleus*, i. *Petrotilapia palingnathos*, and j. *Tropheops macrophthalmus*

In Lake Malaŵi, cichlids have adapted to living in environments that offer a variety of food sources and have evolved specialized morphological structures and feeding behaviors to utilize these sources (Figure 1). A large range of feeding specializations is present in the haplochromines of the lake. The term “explosive radiation” refers to their rapid speciation and the evolution of the staggering number of variations in feeding strategies found in the cichlids of the great lakes of Africa (Fryer & Iles, 1972). In Lake Malaŵi, cichlids feed on a diverse diet including algae, plankton, crustaceans, snails, other invertebrates, scales, fins, whole fishes, and their eggs (see Fryer & Iles, 1972; Ribbink et al., 1983). Nevertheless, astonishingly, these specialized species for the most part are opportunistic and will readily eat anything available. During plankton blooms, which frequently occur in the lake, species with the most diverse feeding specializations congregate in the water column to feed on the plankton. Algae‐feeding mbuna (a group of small rock‐dwelling cichlids) react eagerly to pounded fish flesh bait. When the stomach contents of freshly caught cichlids are analyzed, there can be an astonishing similarity regardless of species and feeding specializations. These cichlids are extremely plastic when it comes to their feeding repertoire (Stauffer & Van Snik‐Gray, 2004).

A possible explanation of the apparent discrepancy between the presence of these precise feeding adaptations on the one hand and the seemingly opportunistic feeding behavior of different species on the other is that these highly defined feeding specializations developed during periods when a severe paucity of food threatened their survival, and currently are important for survival when abundant food is not available (e.g., during the dry season when there is a lack of nutrients). Freshwater lakes with a diverse substrate and inhabited by a varied palette of invertebrate life were most likely present throughout the millennia, and the environmental challenges facing a cichlid in present Lake Malaŵi are expected to be very similar to those faced by its ancestors 50 million years ago. Morphological adaptations that evolved a long time ago are likely stored in every cichlid's genome and under certain circumstances past structural innovations can be “recalled” whenever the environment again requires it.

The stunning similarity of the mouth structure in *Petrochromis* Boulenger, endemic to Lake Tanganyika, and in *Petrotilapia* Trewavas, endemic to Lake Malaŵi was proven to be convergence on the basis of molecular studies (Kocher et al., 1993). The genes responsible for the particular configuration of the flexible teeth and tooth pads likely originated in a common ancestor of the Tanganyika and Malaŵi radiation and were again recruited when the environment included algal growth on rocky substrates.

A study by Colombo et al. (2012) in which the morphology and genetic background of a thick‐lipped species in Lake Tanganyika and in Lake Nicaragua were compared, suggested that a similar set of genes was recruited to develop the hypertrophied lips in the Central American and in the African species. These two thick‐lipped cichlids belong to two different subfamilies of Cichlidae that are at least 50 million years apart.

Historically, observed behavioral characters have been used to diagnose species and used as synapomorphies to describe new genera from Lake Malaŵi (see Stauffer et al., 1993, 1995). For example, when Konings and Stauffer (2006) expanded the diagnoses of a genus of rock‐dwelling cichlids from Lake Malaŵi, *Metriaclima,* they used a behavioral character of these species, that is, feeding at a 90° angle to the substrate, as a synapomorphy. They associated this feeding behavior with the fact that these fishes could abduct their jaws 180°. Similarly, the jaw shape of the paedophage *Caprichromis liemi* was associated with the observed behavior of ramming mouthbrooding cichlids to feed on the eggs and larvae expelled from the mouth. Although these behaviors can be recorded for shallow‐water species, to date it is not feasible to observe such behaviors for deep‐water or very rare species. Stauffer and McKaye (1986) used jaw morphology to postulate that *Diplotaxodon greenwoodi*, a deep‐water cichlid from Lake Malaŵi, was a paedophage.

The varied morphologies found in cichlids from Malaŵi are best viewed using high‐resolution X‐ray computed tomography (HRCT). The use of such technology is nondestructive, so we can collect data on type specimens from museum collections. For example, the description of *Metriaclima zebra* (Boulenger) was based on a single specimen deposited in the British Museum of Natural History (BMNH 1891.12.17.7). High‐resolution X‐ray computed tomography permitted us to examine the internal structure of its head and to determine the various synapomorphies. Furthermore, we can view the internal morphology in increments of 20–35 µm and examine areas that would otherwise be destroyed by dissection. This technology can be applied to similar phenotypes of cichlids found in other lakes, even on different continents. The purpose of this paper is to illustrate how observed behavioral traits of shallow‐water species can be linked with morphological attributes using data collected from selected cichlids from Lake Malaŵi using the aforementioned techniques. Such associations can be used to predict behavior of deep‐water or rare species based on head morphology.

## 2 METHODS

**FIGURE 2:**
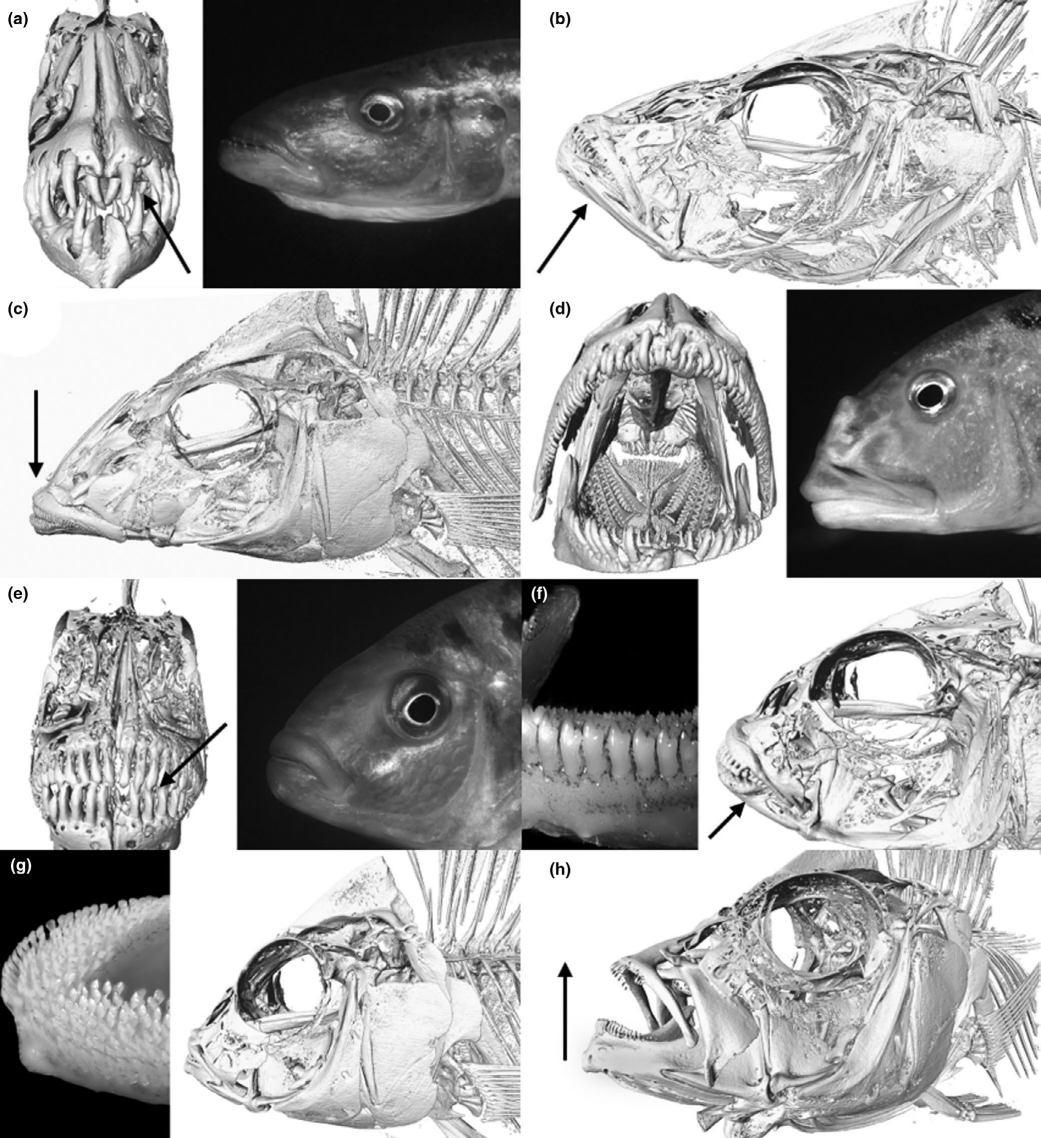
High‐resolution (0.035 mm) microCT scans and photographs of piscivorous fishes with key characters emphasized by an arrow: (a) large pointed teeth of *Rhamphochromis esox* (PSU‐A‐487), (b) protrusible mouth *Dimidiochromis compressiceps* (PSU‐A‐089), (c) downward‐projecting snout of *Nimbochromis linni* (PSU‐A‐101), (d) *Aristochromis christyi* (PSU‐A‐2506), (e) large conical teeth of *Docimodus evelynae* (PSU‐A‐249), (f) heavy lower jaw of *Genyochromis mento* (PSU‐A‐007), (g) numerous teeth of *Corematodus taeniatus* (PSU‐A‐762), and (h) upturned jaw of *Protomelas krampus* “paedophage” (PSU‐A‐776)

The specimens referred to herein were collected by SCUBA divers who chased them into a monofilament net. They were anesthetized with clove oil (IACUC 110784), preserved in 10% formalin, and then placed in 70% ethanol for permanent storage in the Penn State University Fish Museum. After preservation, they were scanned on the high‐resolution X‐ray computer system in the Center for Quantitative X‐Ray Imaging (CQI) at Penn State University. Specimens were mounted vertically, the mandibles pinned together to create a standard position, and scanned with target pixel and slice resolutions of approximately 35 µm. Scan data were reconstructed as 16‐bit TIFF images with a 1,024 × 1,024 pixel grid. For each individual, the entire head was scanned. The volumetric image datasets for each fish were used to create a three‐dimensional isosurface reconstruction (Figure 2) in order to study bone tissue using the visualization software Avizo 9.3 (VSG). Because all of the fishes were HRCT scanned with the same energy settings and voxel resolutions, a global threshold was used for all datasets to separate bone from nonbone for the three‐dimensional reconstructions. All data referenced herein have been uploaded to morphosource.org and are globally available. In many cases, only a single specimen was scanned. Specimens to be scanned were chosen based on their size and availability. When multiple scans were available as a result of other studies, a “typical” specimen was chosen.

## 3 RESULTS

### 3.1 Piscivores

Most piscivores have large mouths and jaws set with large unicuspid teeth, but paedophages, which feed on eggs and larvae that they steal from the mouth of a mouthbrooding female, have small teeth that are embedded in the gums. Other piscivores that mainly target fry or juvenile fishes also have no need for large teeth to hold a wriggling prey. All piscivores have slender, unicuspid teeth on the pharyngeal jaws.

*Rhamphochromis esox* (Boulenger; PSU‐A‐487; Figure 2a). The open water of Lake Malaŵi is home to several pursuit hunters that have a streamlined body allowing them to swim with burst speed snatching smaller prey. All the species in *Rhamphochromis* Regan are pursuit hunters. Most of them range hundreds of meters offshore but a few occur in shallow bays. The mouth of these piscivores is characteristically set with large, pointed teeth that are set widely apart. Prey is normally seized at the head as it needs to be swallowed head‐first. If prey is caught mid‐body, it is manipulated so that it can be swallowed head‐first.

*Dimidiochromis compressiceps* (Boulenger; PSU‐A‐089; Figure 2b) is a piscivore that is built to conceal itself among reed stands and in *Vallisneria* beds. Its silvery body is extremely laterally compressed and its back bears dark green stripes. When lying in ambush, it positions itself head‐down among the reeds or *Vallisneria*, where its green stripes camouflage it and waits motionless for small fishes to pass. Prey are quickly seized and sucked inside the protrusible mouth.

*Nimbochromis linni* (Burgess & Axelrod; PSU‐A‐101; Figure 2c) is specialized for snatching young mbuna out of cracks between rocks. Its characteristic behavior is not found in any other known predatory cichlid of Lake Malaŵi. The large size of *N. linni* (maximum 30 cm) prevents it from penetrating the hideaways of the small mbuna, but after having located a site with small fish it slowly drops down onto a rock with its characteristic downward‐projecting snout at the edge of it, above the crevice. Its mottled pattern blends well with the environment while it remains motionless for several minutes stealthily observing the small mbuna hiding in the crack. As soon as one of them comes within reach of the very protractile mouth, it is sucked out of its shelter. When *N. linni* expands its buccal cavity its protruded mouth functions like the hose of a vacuum cleaner.

*Aristochromis christyi* Trewavas (PSU‐A‐2506; Figure 2d) is not a swift swimmer and has a peculiar manner of attacking prey. The characteristic feature of its hunting behavior is the tilted position of the body. The predator focuses just one eye on its prey and can tilt its body to either side. When it has “eyed” its prey, it slowly descends to approach the victim. Just before it strikes, it shakes its body as if it is a sick fish trying to regain normal position before falling to the bottom. When it comes within striking distance of an unwary prey, a sudden stroke sideways with its head secures the victim between the jaws. *Aristochromis christyi* is specialized in hunting rather large prey, sometimes about a third its own length. The exceptionally strong jaws prevent escape.

Juvenile *Docimodus evelynae* Eccles and Lewis (PSU‐A‐249; Figure 2e) inhabit relatively shallow water and behave as cleaner fishes, screening host‐cichlids for possible parasites or fungus that is present in wounds (Eccles & Lewis, 1976; Ribbink, 1984). When *D. evelynae* has grown to about 60 mm in length, its teeth gradually change as does its feeding behavior. During this change, it feeds from the *aufwuchs* or from the plankton, when available. By a size of about 10 cm, large conical teeth have replaced all the juvenile tricuspid ones, and at about the same time it starts to frequent much deeper regions, now feeding on scales from cichlids and barbs, and pieces of skin from catfishes. *Bagrus meridionalis* Günther, locally known as Kampango, seems to be a victim of these attacks, but attacks on clariid catfishes have also been observed. Most large catfish show scars on the body, evidence of encounters with *D. evelynae*. The difference in the size of *D. evelynae* in the cleaning and predatory stages—smaller than 6 cm versus larger than 10 cm—is apparently sufficient for prospective hosts (or victims) to correlate with the difference in feeding behavior.

*Genyochromis mento* Trewavas (PSU‐A‐007; Figure 2f) is a very common mbuna and the only species that is found in all habitats except the open water column, and at all locations around the lake. It feeds on the fins and scales of other cichlids (Fryer et al., 1955) and is morphologically characterized by a heavy lower jaw. It shows a surprising agility and vigor in its attacks on other fishes. Small fishes, large fishes, even divers’ bare legs—anything that passes by is attacked from below. The preferred sites of attack are the caudal peduncle and fins. Egg‐spots are favored too, and many cichlids have notched anal and caudal fins, evidence of an encounter with *G. mento*. The fin‐biter practices the following technique: it remains in partially hidden, locations and attacks, with lightning‐fast sallies, as many fishes as possible. After a few minutes, most of the sedentary mbuna will have noticed the predator and thereafter avoid passing by or chase it before it can attack. At this point, *G. mento* moves to another site a meter away where it can again attack passing mbuna. Adult *G. mento* seems to prefer attacking fighting mbuna where they collect any scales that are dislodged from the combatants’ flanks. They may also actively join in the combat and feed directly from the flanks of the opponents, while they are concentrating on each other and hardly notice the stealthy approach of *G. mento*. Female cichlids are mutilated to a far lesser extent. The victims are usually mbuna, but larger cichlids are also attacked, especially those in rocky areas where *G. mento* can hide before it attacks. Like most predatory fishes, *G. mento* has a feeding territory, but in this instance, the territory is an area with a radius of about 50 cm centered on the fish and moves with it. Only conspecifics are chased from this territory.

The scale‐eater *Corematodus taeniatus* Trewavas (PSU‐A‐762; Figure 2g) normally attacks sand‐dwelling species that are characterized by a diagonal stripe (Fryer & Iles, 1972; Trewavas, 1947). Like its victims, *C. taeniatus* has a diagonal stripe on its body, which it has evolved as camouflage to render it inconspicuous among the schools of sand‐dwelling cichlids. *Corematodus taeniatus* feeds mainly from the caudal peduncle of its prey and frequently scrapes and dislodges the small scales covering this vulnerable part of its victims. The numerous teeth in its jaws are positioned in wide bands and feel like sandpaper to the touch. It has a lake‐wide distribution and is rather common among schools of sand‐dwellers.

*Protomelas krampus* (PSU‐A‐776; Figure 2h) is a predatory cichlid with an upturned jaw specialized in knocking eggs and larvae out of mouths of mouthbrooding females (McKaye & Kocher, 1983). After the initial impact on the female's head—most of these paedophages but the female's buccal pouch holding the brood from below, but *P. krampus* strikes the female's head coming from above—the brood is quickly gulped by the predator. This happens so fast that it cannot be witnessed by the human eye; however, stomach content analyses revealed that these are full of cichlid eggs and larvae.

### 3.2 Invertebrate predators

Depending on the hardness of the type of prey, the teeth on the pharyngeal jaws of these predators range from slender and pointy to heavy and molar shaped. This trophic group also includes species that have additional adaptations such as thick lips or an enhanced sensory system on the head.

**FIGURE 3:**
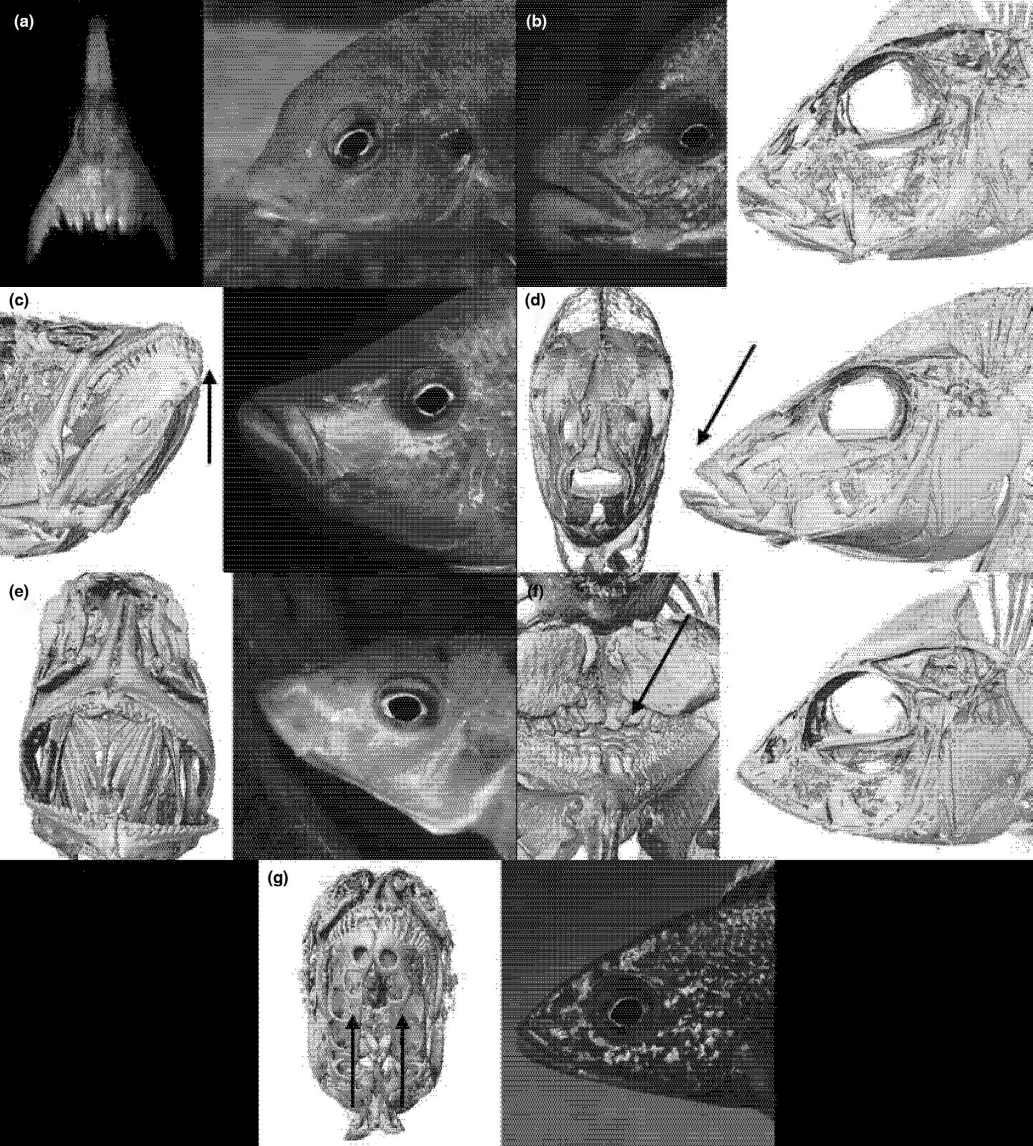
High‐resolution (0.035 mm) microCT scans and photographs of invertivorous fishes with key characters emphasized by an arrow: (a) long pincer‐like teeth of *Labidochromis maculicauda* (PSU‐A‐213), (b) fleshy lips of *Placidochromis milomo* (PSU‐A‐057), (c) upturned mouth of *Caprichromis liemi* (PSU‐A‐1807), (d) long snout of *Taeniolethrinops praeorbitalis* (PSU‐A‐2083), (e) downward protrusible mouth of *Ctenopharynx pictus* (PSU‐A‐757), (f) thick pharyngeal bones of *Trematocranus placodon* (PSU‐A‐039), and (g) enlarged sensory pits of *Aulonocara stuartgranti* (PSU‐A‐2873)

*Labidochromis maculicauda* Lewis (PSU‐A‐213; Figure 3a) feeds on insects and crustaceans that hide in dark recesses of the rocky habitat. It has a very light and conspicuous coloration but is nevertheless small and usually occurs in solitary. It wanders through the habitat, never lingering at any particular spot. It is tolerated in the territories of most other species and prefers dark caves, the ceilings of which are visually inspected for chance prey. The teeth of *L. maculicauda* are long and pointed and act like pincers tweezing prey out of tiny cracks and pockets in the rocky surface (Lewis, 1982). Prey is located visually and the fish, therefore, needs to search in sediment‐free environments as on sediment‐covered rocks prey is virtually invisible.

Several cichlid species are characterized by the possession of hypertrophied lips. The thick‐lipped cichlids of Lake Malaŵi can be divided into two groups, one containing species with thickened lips, which feed predominantly from cracks and slits between rocks (e.g., *Protomelas ornatus* (Regan)); and another consisting of species with very large, rubbery lips. *Placidochromis milomo* (Oliver; PSU‐A‐057; Figure 3b) is a member of this second group and likewise feeds from rocky substrates, from small pockets in the rocks rather than cracks and slits. Small invertebrates, the main food source for rubber‐lipped species, find shelter in the rough surface of the rocky substrate. The predator carefully selects the site of attack before striking the surface of the rock in order to extract the prey. If the site is perceived to be of nutritious value, it will quickly bite. The lips of these species are very swollen and jellylike to the touch. When the fish strikes the substrate these lips are smashed against the rock and seal, like a gasket, the hole in which the prey has hidden itself (preventing it from escaping). When the buccal cavity is subsequently expanded the prey is efficiently sucked out of its shelter. When the sediment layer covering the aufwuchs is thick, a cloud of dust accompanies each bite.

Apart from some small species that clean parasites from larger fish, *Caprichromis liemi* (McKaye & Mackenzie; PSU‐A‐1807; Figure 3c) appears specialized in picking fish lice (*Argulus africanus*) from other cichlids (Konings, 1991). The African fish louse is rather common on the throat and breast region of many cichlids and with a diameter of about 5–10 mm. *Caprichromis liemi* positions itself about 50 cm below the louse‐carrying fish and with a lightning‐fast dart latches its upturned mouth on to a louse which is then dislodged by a twist of the body. If *C. liemi* removes lice from throats of mouthbrooding females, these may actually release a part of their brood upon impact. Eggs and larvae are extremely nutritious and preferred by all fish over hard‐shelled invertebrates. The lice‐removing behavior may in fact have triggered the paedophagy employed by a number of species in Lake Malaŵi (see above). *Caprichromis liemi* also actively chases mouthbrooding females and feeds on their broods.

The members of *Lethrinops* Regan and similar genera find their food, insects and crustaceans, in the sand. Such prey is normally not visible and must be extracted from the sand by random scooping and sifting (Fryer & Iles, 1972). These cichlids are very common, and the sandy lake bottom is often pock‐marked from their digging activities. Some species, such as *Taeniolethrinops praeorbitalis* (Regan; PSU‐A‐2083; Figure 3d), have very long snouts and can thus scoop sand from deeper layers than the smaller species with short snouts.

The sediment‐covered substrate is rich in microorganisms and debris which are eaten by small crustaceans. *Ctenopharynx pictus* (Trewavas; PSU‐A‐757; Figure 3e), a peculiar cichlid, is specialized in feeding on these small invertebrates (Ribbink et al., 1983). This three‐spotted species has a lake‐wide distribution and has a very protrusible mouth, which opens downward. The expanded gape, which is almost perpendicular to the body, is held a few millimeters above the substrate and the buccal cavity is then extended by opening the gill covers wide. As a result, a stream of water is sucked into the mouth. Largely because of the wide gape, only the small and free‐moving invertebrates are sucked into the mouth, while most of the actual sediment remains on the rocks. *Ctenopharynx pictus* literally vacuum‐cleans the algal carpet. To prevent the food‐particles thus collected from escaping via the gills, the anterior gill arches each bear a set of no less than 35 rakers.

*Trematocranus placodon* (Regan; PSU‐A‐039; Figure 3f) has small teeth and weak oral jaws, reminiscent of species of the genus *Lethrinops*, and these certainly do not suggest a habitual snail‐crusher, which it is (Fryer & Iles, 1972). The teeth on the pharyngeal bones are very thick and can withstand the pressure when a snail is crushed between the bones. The favorite type of snail of *T. placodon* is *Bulinus nyassanus* but it will also feed on *Melanoides tuberculata* (Evers et al., 2006). These snails live in the upper layer of the sand, normally hidden from view. The enlarged sensory pores on the lower half of the head are very sensitive in detecting the tiny movements snails make in the sand. *Trematocranus placodon* normally hovers about 20 cm over the bottom and waits until it hears something moving in the sand before it dashes forward scooping up a mouthful with the prey. The snails of interest are usually not larger than about 1 cm in diameter, and they are not completely pulverized between the pharyngeal jaws—just cracked so that the gastric juices can digest the shell's contents. In fact, some of the smaller snails are swallowed whole.

All members of *Aulonocara* Regan are characterized by an enlarged lateral line system (the sensory system), in particular on the head (Konings, 1989). This extension, which makes them much more perception‐sensitive, is clearly visible externally as pits and grooves, especially on the lower part of the head. All cichlids equipped with such enhanced sensors use these organs as sensitive food‐detectors. The natural behavior of *Aulonocara stuartgranti* Meyer and Riehl (PSU‐A‐2873; Figure 3g) provides the vital clue to the purpose of the sensory enhancement on the head (Konings, 1989). Territorial males, as well as females, and juveniles all hover about a centimeter above the sandy substrate barely moving a fin. The fish remain stationary over their feeding grounds and their “trance” is only interrupted by an occasional plunge of the snout into the sand. The enlarged sensory pits which are located on the lower part of the jaw, register with high sensitivity the minute movements of crustaceans and snails hidden in the sand. As soon as a moving prey item is detected, a quick bite in the sand secures it inside the predator's mouth. By churning, the fish separates the prey from the mouthful of sand and either expels the latter through the gills or spits it out.

### 3.3 Herbivores

Most cichlid species in Lake Malaŵi are herbivores feeding off the rich algal layer that covers rocks—the so‐called aufwuchs—and which is also found on plants and other substrates. The aufwuchs consists of a variety of algae: the tough strands of some filamentous algae (*Calothrix* and *Cladophora*) are attached to the rocks and form the matrix on which other algae, the so‐called “loose aufwuchs,” grow. The loose aufwuchs contains different types of algae strands, but the many short strands of blue–green algae (Cyanobacteria) and the unicellular algae (diatoms) constitute its most nutritious part.

Those mbuna that are mostly true herbivores have numerous fine teeth on the pharyngeal bones. The upper and lower pharyngeal teeth are rubbed against each other and partially crush the tough green algae between them. Nevertheless, a lot of the green algae remain intact and leave the body as a kind of indigestible fiber. To allow the digestive juices enough time to penetrate the broken algal cells, the intestine of herbivores is considerably longer than the fish itself (up to ten times longer). Typically, the lining of the body cavity in herbivores is pitch‐black.

**FIGURE 4:**
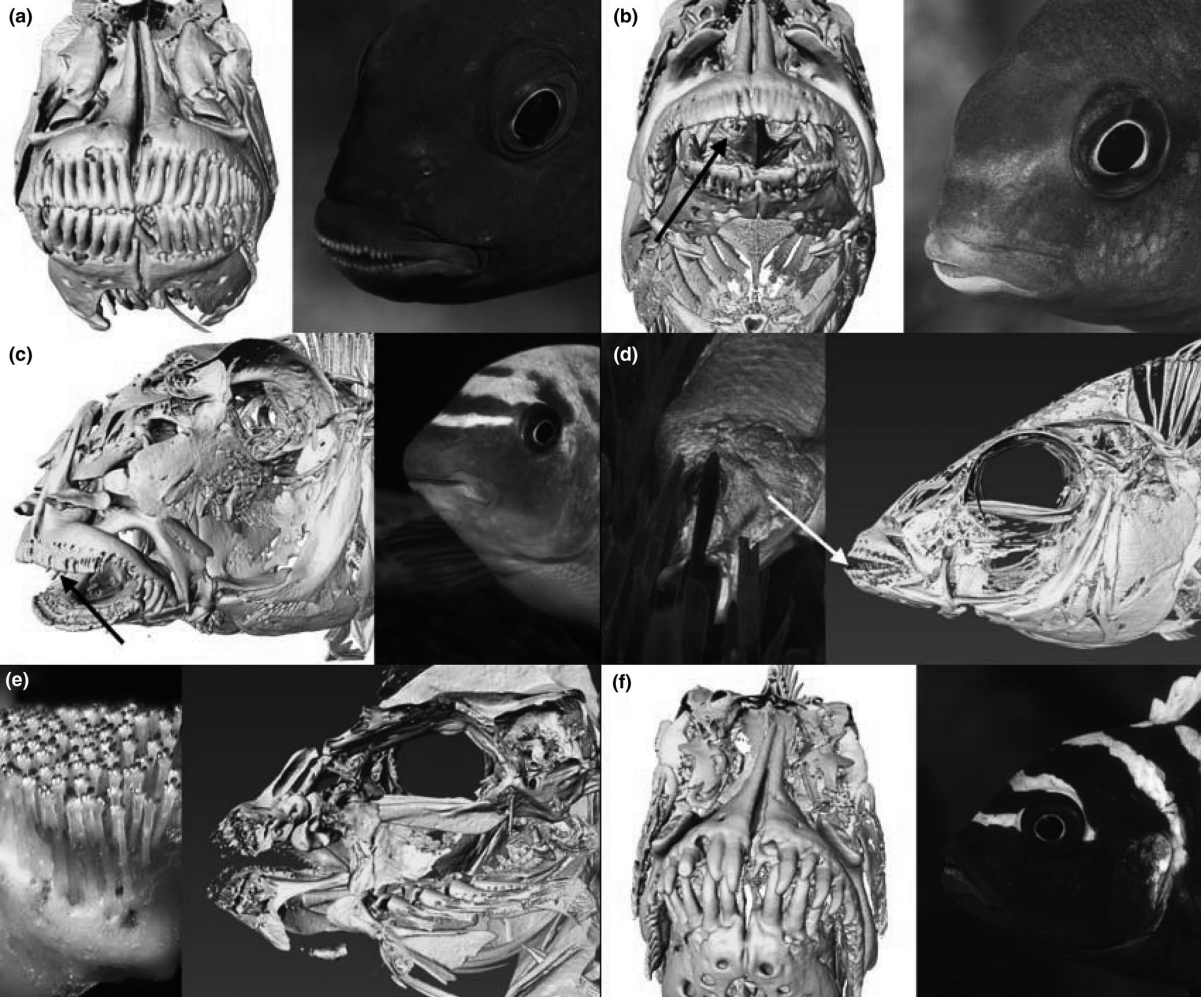
High‐resolution (0.035 mm) microCT scans and photographs of herbivorous fishes with key characters emphasized by an arrow: (a) strong jaws of *Chindongo bellicosus* (PSU‐12576), (b) small close‐set teeth of *Tropheops tropheops* (PSU‐A‐257), (c) straight‐line mouth of *Labeotropheus fuelleborni* (PSU‐12923), (d) deeply set teeth of *Hemitilapia oxyrhynchus* (PSU‐A‐573), (e) comb‐like triple crown teeth of *Petrotilapia nigra* (PSU‐4803), and (f) widely spaced conical teeth of *Cynotilapia zebroides* (PSU‐A‐054)

*Chindongo bellicosus* Li, Konings, & Stauffer (PSU‐12576; Figure 4a) has very strong jaws and can grip the aufwuchs and tear pieces from it by swimming away with a mouthful. The algal layer, however, is normally strongly anchored to the substrate and algal strands, the matrix of the mat, too tough for tearing so that only small bits and pieces are removed per bite. This manner of feeding is not very effective and as a result species employing such technique are usually small and/or aggressive in defending their feeding territories. *Chindongo bellicosus* is so energetically aggressive that it is able to keep all other grazers from its premises, thereby creating a so‐called algal garden, that is, a distinctly visible yellowish spot on the substrate where algae are grown abundantly providing enough food for the tenant.

The members of the genus *Tropheops* Trewavas are almost all found in the upper rocky habitat free of sediment and have very strong jaws with small, closely‐set teeth. The teeth of upper and lower oral jaw of *Tropheops tropheops* (Regan) neatly fit into each other and have a very strong hold on the algal strands when it bites in the algal mat. While holding tight on the algae, it rapidly shakes its entire body and thus dislodges or tears off the strands. In the very shallow rocky habitat, the algae are also tougher and *T. tropheops* (PSU‐A‐257; Figure 4b), found in these shallow areas, has a very short lower jaw (and concomitantly a prominate curved head), which increases its grip on the algae.

*Labeotropheus fuelleborni* Ahl (PSU‐12923; Figure 4c) is characterized by a fleshy “nose,” which overhangs the mouth. When it is held upside‐down the mouth is seen to be a straight line across the full width of the head. Its ventral position allows *L. fuelleborni* to feed in a position almost parallel to the rock, its body making an angle of approximately 30° with the substrate. It thus remains in close contact with the substrate while cropping algae. Both nose and chin are callused, probably as a result of continuous contact with rough substrates during feeding. The effect of the fish closing its mouth on the firmly attached filamentous algae is to pull itself closer to the substrate and the nose then functions as a pivot, allowing the fish to pull off the algae by leverage rather than energy‐consuming jerking of the body. This not only saves energy but also allows *L. fuelleborni* to remain in close contact with the rocks, thus reducing the risk of being swept away by the turbulent water. Moreover, it allows greater quantities and more tightly attached, algae to be cropped using the three or more rows of tricuspid teeth in the outer jaws; this feeding method is so efficient that the algae are removed completely, leaving visible scrape marks in the biocover.

*Hemitilapia oxyrhynchus* Boulenger (PSU‐A‐573; Figure 4d) is specialized in scraping algae from *Vallisneria* leaves but is seen feeding from other plants as well (Fryer & Iles, 1972). It does not eat the leaves, but just “cleans” them of attached algae (and possibly invertebrates). Its feeding technique is very characteristic and can be readily appreciated when observing these cichlids in the lake. The leaves are scraped from bottom to top, and the fish turns on its side before grasping the base of a leaf. It then slides the leaf through its mouth by jerking three or four times. When the leaves are thickly covered with aufwuchs, *H. oxyrhynchus* can also be observed slowly nibbling its way to the top (of the leaf). The teeth of the outer jaws are situated deep inside the mouth, especially along the sides. As the leaves are scraped, the aufwuchs accumulates in the space between the teeth and the lips.

*Petrotilapia nigra* Marsh (PSU‐4803; Figure 4e) feeds on loose aufwuchs. It feeds at almost perpendicular angles to the substrate and is able to align the teeth of both upper and lower jaws in the same plane by abducting its jaws to a 180° angle opening. The jaws are then pressed against the substrate and closed. While closing the mouth, the teeth rake through the algal matrix anchored to the substrate and collect loose material, consisting mainly of diatoms in the upper rocky habitat. Members of the genus *Petrotilapia* are characterized by broad, fleshy lips densely covered with long, slender, and flexible teeth with a three‐pointed crown. The teeth, which are permanently exposed even when the mouth is closed, are excellent tools for combing loose aufwuchs. The large mouth and the numerous teeth efficiently collect the fine, loose material. The very flexible teeth merely comb the algae strands collecting diatoms and blue–green algae.

The peculiarity of *Cynotilapia zebroides* (Johnson; PSU‐A‐054; Figure 4f) is that it feeds predominantly on miniscule phytoplankton (Genner et al., 1999) but has large, widely spaced conical teeth. Most mbuna feed on plankton when available in abundance but *C. zebroides* feeds predominantly in mid‐water continuously picking at tiny food morsels that are mostly invisible to the human eye. The food is sucked in, and in principle, the species would not need strong jaws, but they do have heavy jaws. Of course, large canines need a firm base and that may be the reason for the relatively strong jaws. Plankton can, in fact, be collected even without teeth, but males may need sharp teeth for territorial defense.

### 3.4 Explosive radiation of Lake Malaŵi Cichlids

Although the haplochromines of Lake Malaŵi most likely evolved from a single ancestor (Genner et al., 2015) over a relatively short evolutionary time scale, they represent an extraordinary array of ecological adaptations. It was suggested (Liem, 1973) that the exceptional diversity of cichlids, in general, is a result of the versatile pharyngeal apparatus, in particular the independent movement of the upper and lower pharyngeal jaws. The versatility of the pharyngeal jaws permits the efficient handling of many different prey items, and the resultant flexibility allows for a more varied phenotypic response to changing requirements. The enhanced functional flexibility of cichlids not only provides phenotypic plasticity, but also means that only small genetic changes are necessary to change feeding specialization. For example, the short lower jaw of *Labeotropheus fuelleborni*—compared with that of *Metriaclima zebra*—is caused by a difference in timing during development and associated with a point mutation in a transcriptional regulator (Powder et al., 2014).

Apart from the explosive radiation that has occurred among the haplochromines in Lake Malaŵi (Fryer & Iles, 1972), “explosive” speciation resulted into hundreds of species endemic to areas that were dry ground less than 25,000 years ago when the water level was about 150 m below current (Lyons et al., 2011, 2015). In particular, the rock‐dwelling haplochromines, the mbuna, have evolved in a series of algae‐harvesting specialists, the characteristics of which have been used to classify them in the various genera. A member of each of the different herbivore guilds—*Labidochromis* Trewavas, *Metriaclima* Stauffer, Bowers, Kellogg, and McKaye, *Tropheops*, *Pseudotropheus* Regan, *Chindongo* Li, Konings and Stauffer, *Labeotropheus* Ahl, and *Melanochromis* Trewavas*—*is present at every shallow rocky habitat around the lake. New populations of each of these guilds were founded at virtually all noncontinuous rocky habitats that became submerged during a lake level rise, and many speciated (Won et al., 2005). Consequently, most if not all of these new forms were again lost when the lake retreated to a lower stand.

Besides the many feeding specialists in Lake Malaŵi, there is of course also a group of species that developed behavioral adaptations to increase the efficiency of obtaining available resources. The paedophages discussed above basically belong to this group but also the famous sleeper, *Nimbochromis livingstonii* (Günther), a species who pretends to be dead in order to attract its food: inexperienced juvenile fish that appear to be attracted to the predator's unique color pattern.

Delineation of cichlid fishes from Lake Malaŵi has been challenging in part because many of these species are morphologically similar. The first attempts to sort the haplochromines from Lake Malaŵi involved the shape of tooth and jaw structures (Regan, 1922; Trewavas, 1935). The morphology of teeth and jaws was replaced by melanin patterns and coloration when Eccles and Trewavas (1989) recognized them to be more reliable characters to distinguish taxa. Later, cranial morphology played again a role in taxonomy of fishes from Lake Malaŵi when Stauffer et al. (1997) used the angle of the ethmo‐vomerine complex to delineate the genus *Metriaclima* and recently by Li et al. (2016) to define the genus *Chindongo*.

## 4 DISCUSSION

The main morphological characteristics that unite cichlid species into trophic guilds are based on the shape of the pharyngeal jaws, the structure of their teeth, and the size and shape of the dentary. Piscivores from Lake Malaŵi commonly have a slender, elongated lower pharyngeal jaw set with laterally compressed, acutely pointed teeth. Piscivores that need to secure moving prey between their jaws—ram feeders—have relatively large, unicuspid teeth in the oral jaws. The lower jaw in such predators is usually strong, heavy, and prognathic. Other piscivores that feed on smaller items such as eggs, fry, fins, or scales usually have smaller oral teeth that are usually bicuspid and numerous.

The herbivores conversely have a wide lower pharyngeal jaw set with minute teeth with a rounded tip. The precise function of these teeth is unknown, and they do not seem to mechanically alter the algae (Cyanobacteria), which is the main item consumed by all herbivores of Lake Malaŵi. They may, however, concentrate and bundle the various stands of algae before they are swallowed. There are two main types of herbivores, pickers, and combers. The common morphological characteristic of the algae pickers is a short dentary that gives the fish an overbite and closely packed oral teeth each with two or three short tips. It appears that the species with the shortest dentary (e.g., *Labeotropheus* spp.) have the firmest grip on the blue–green algae and can thus crop the shortest strands. The algae‐combing herbivores normally have terminal mouths with wide tooth bands in both upper and lower jaws. The bi‐ and tricuspid oral teeth have relatively long tips that act as the tines of a fork or comb, raking the algal matrix collecting the loose strands of algae.

The invertivores appear to have little in common when it comes to the morphology of oral and pharyngeal jaws although many have enlarged teeth in the pharyngeal jaws. While the herbivores are suction feeders and the piscivores predominantly ram feeders, the invertivores include ram, suction, as well as protrusion feeders. Invertebrates have been the quintessential food of most species of cichlids and the array of feeding mechanisms employed by invertivores probably have evolved long before they invaded Lake Malawi, with a possible exception of the sonar‐feeding *Aulonocara* spp., which seem to be unique to the lake.

The above examples highlight the use of high‐resolution X‐ray computed tomography to link morphological character states with observed behaviors. For many of the cichlids in Lake Malaŵi, the species is known only from the holotype. Additionally, the selected cichlids from Lake Victoria are only known from museum specimens since many are no longer found in the lake (Kishe‐Machumu et al., 2015; Seehausen et al., 1997). High‐resolution X‐ray computed tomography will permit scientists to infer life history and behavior characters (i.e., functional morphology) of rare or extinct taxa from a detailed examination of morphology and linkages between morphology and behavior found in extant species. Finally, computed tomography will permit us to quantify skeletal features to determine both sympleisiomorphic and synapomorphic character states.

## CONFLICT OF INTEREST

None of the authors have a conflict of interest relative to this manuscript.

## AUTHOR CONTRIBUTION

**Adrianus F Konings:** Conceptualization (supporting); Data curation (supporting); Resources (supporting); Visualization (lead); Writing‐original draft (lead). **Joshua Michael Wisor:** Data curation (equal); Formal analysis (equal); Methodology (supporting); Resources (equal); Software (lead). **Jay Stauffer:** Conceptualization (equal); Funding acquisition (equal); Methodology (equal); Resources (equal); Supervision (equal); Visualization (equal); Writing‐review & editing (equal).

## ETHICAL APPROVAL

All fishes were anesthetized with clove oil (IACUC 110784), preserved in 10% formalin, and then placed in 70% ethanol for permanent storage in the Penn State University Fish Museum.

## Data Availability

All scan data referenced herein have been uploaded to MorphoSource.org (www.morphosource.org) and are globally available.
